# Supplementary material for: Modeling glioblastoma heterogeneity as a dynamic network of cell states
Source: Mol Syst Biol. 2021 Sep 16;17(9):e10105. doi: 10.15252/msb.202010105 (PMC8444284; doi:10.15252/msb.202010105)
Supplement: Supplementary file 6 — Source Data for Figure 5 [file MSB-17-e10105-s004.zip › Figure5A_sourcedata/GSEA_3017/hallmarks_stateC.GseaPreranked.1621934520804/HALLMARK_ESTROGEN_RESPONSE_LATE.html]

Details for gene set HALLMARK\_ESTROGEN\_RESPONSE\_LATE[GSEA]

|  || Dataset | state13017 |
| Phenotype | NoPhenotypeAvailable |
| Upregulated in class | na\_neg |
| GeneSet | HALLMARK\_ESTROGEN\_RESPONSE\_LATE |
| Enrichment Score (ES) | -0.38111174 |
| Normalized Enrichment Score (NES) | -1.6849653 |
| Nominal p-value | 0.02685624 |
| FDR q-value | 0.051320307 |
| FWER p-Value | 0.279 |
Table: GSEA Results Summary

  

Fig 1: Enrichment plot: HALLMARK\_ESTROGEN\_RESPONSE\_LATE      
 Profile of the Running ES Score & Positions of GeneSet Members on the Rank Ordered List

  

| PROBE | GENE SYMBOL | GENE\_TITLE | RANK IN GENE LIST | RANK METRIC SCORE | RUNNING ES | CORE ENRICHMENT || 1 | TOP2A |  |  | 52 | 0.426 | -0.0432 | No |
| 2 | GINS2 |  |  | 83 | 0.360 | -0.0555 | No |
| 3 | CKB |  |  | 102 | 0.335 | -0.0498 | No |
| 4 | ASCL1 |  |  | 145 | 0.290 | -0.0906 | No |
| 5 | RBBP8 |  |  | 177 | 0.267 | -0.1150 | No |
| 6 | FDFT1 |  |  | 243 | -0.262 | -0.1988 | No |
| 7 | MYOF |  |  | 251 | -0.263 | -0.1818 | No |
| 8 | ABHD2 |  |  | 296 | -0.275 | -0.2279 | No |
| 9 | DHCR7 |  |  | 322 | -0.285 | -0.2397 | No |
| 10 | MEST |  |  | 352 | -0.299 | -0.2570 | No |
| 11 | UGDH |  |  | 360 | -0.301 | -0.2359 | No |
| 12 | FOS |  |  | 377 | -0.309 | -0.2295 | No |
| 13 | FGFR3 |  |  | 403 | -0.322 | -0.2374 | No |
| 14 | RAB31 |  |  | 445 | -0.349 | -0.2700 | No |
| 15 | PRSS23 |  |  | 510 | -0.438 | -0.3327 | Yes |
| 16 | CD9 |  |  | 515 | -0.443 | -0.2906 | Yes |
| 17 | CA2 |  |  | 518 | -0.448 | -0.2445 | Yes |
| 18 | SNX10 |  |  | 551 | -0.521 | -0.2425 | Yes |
| 19 | CD44 |  |  | 575 | -0.659 | -0.2095 | Yes |
| 20 | CA12 |  |  | 586 | -0.717 | -0.1477 | Yes |
| 21 | FABP5 |  |  | 596 | -1.477 | -0.0000 | Yes |
Table: GSEA details [plain text format]

  

Fig 2: HALLMARK\_ESTROGEN\_RESPONSE\_LATE: Random ES distribution      
 Gene set null distribution of ES for **HALLMARK\_ESTROGEN\_RESPONSE\_LATE**
